# Evaluating the Acute Effect of Stereoscopic Recovery by Dichoptic Stimulation Using Electroencephalogram

**DOI:** 10.1155/2020/9497369

**Published:** 2020-04-13

**Authors:** Wei Shi, Luyang He, Bin Lv, Li Li, Tongning Wu

**Affiliations:** ^1^Department of Ophthalmology, Beijing Children's Hospital, Capital Medical University, Beijing, China; ^2^China Academy of Information and Communications Technology, Beijing, China

## Abstract

Amblyopia is a common developmental disorder in adolescents and children. Stereoscopic loss is a symptom of amblyopia that can seriously affect the quality of patient's life. Recent studies have shown that the push-pull perceptual learning protocol had a positive effect on stereoscopic recovery. In this study, we developed a stereoscopic training method using a polarized visualization system according to the push-pull protocol. Dichoptic stimulation for 36 anisometropic and amblyopic subjects and 33 children with normal visual acuity (VA) has been conducted. Electroencephalogram (EEG) was used to evaluate the neurophysiological changes before, during, and after stimulation. For the anisometropic and amblyopic subjects, the statistical analysis demonstrated significant differences (*p* < 0.01) in the beta rhythm at the middle temporal and occipital lobes, while the EEG from the normal VA subjects indicated no significant changes when comparing the results before and after training. We concluded that the dichoptic training in our study can activate the middle temporal visual area and visual cortex. The EEG changes can be used to evaluate the training effects. This study also found that the beta band EEG acquired during visual stimulation at the dorsal visual stream can be potentially used for predicting acute training effect. The results facilitated the optimization of the individual training plan.

## 1. Introduction

Amblyopia is a common developmental visual disorder [1], which reduces the visual acuity (VA) of one or both eyes without obvious defects in the visual pathway, and cannot be solved immediately using eyeglasses or contact lenses [2]. Patients with amblyopia usually present several visual functional deficiencies, such as refractive errors, low sensitivity to contrast or dynamic objects, and limited stereopsis [3, 4], which is estimated to affect 1-5% of the adult population and even more for children [2, 4]. Stereoscopic vision is one of the most advanced visual functions, which provides a sense of depth in the environment and helps develop basic skills, such as grasping, catching, and walking around obstacles at a high speed [5]. Hence, poor stereoscopic depth perception can seriously affect the quality of patient's life. Therefore, it has clinical importance to develop treatment methods for the recovery of stereopsis.

The conventional methods for stereoscopic recovery included monocular occlusion therapy and therapeutic drugs such as atropine [2, 6, 7]. These methods cover or blur the sound eye to force the use of the amblyopic one [8], with the hypothesis that shielding the function of the strong eye facilitated the development of the weak eye. As consequence, the disparity of VA would be levelled. These treatments are often exclusive to children, and adults with stereoscopic deficits are considered untreatable [2]. The use of monocles has been reported to help 50% to 85% of amblyopia children achieve normal VA [6], but with a significant recurrence rate as high as 27% [9]. Dichoptic treatment is another effective approach to solve stereoscopic vision problems [2]. This treatment presents different images to both eyes simultaneously, aiming to reduce intraocular suppression, which is the primary cause for multiple visual deficits [10]. Based on this concept, a series of dichoptic perceptual learning methods became available, including dichoptic videos [11, 12], three-dimensional (3D) video games [13, 14], and virtual reality systems [15, 16]. These studies suggested that VA and stereo sensitivity can be improved through 5 to 30 hours of perceptual learning sessions for children and adults [14, 15]. Among the dichoptic perceptual learning methods, Ooi et al. [17] proposed a push-pull perceptual learning protocol. This protocol can effectively reduce sensory eye dominance and enhance binocular balance [17, 18]. It was designed to stimulate the weak eye (push), while suppressing the perception of the strong eye (pull) in order to recalibrate the binocular balance of excitatory and inhibitory interactions. In contrast, the conventional push-only protocols solely stimulated the weak eye without inhibiting the strong one. This treatment shifted the balance between two eyes towards the weak one and thus improved stereopsis. Numerous studies indicated that this protocol would be promising for stereoscopic recovery [2, 18, 19].

The widely used methods for evaluating stereoscopic recovery were based on clinical examinations, e.g., synoptophore examination, Randot circles test, and Titmus test [20]. These tests focused on assessing stereo sensitivity through behavioural reactions, which may depend on subjective description [2]. Moving toward an objective evaluation for stereoscopic recovery, recent studies have implemented neurophysiologic methods such as functional magnetic resonance imaging (fMRI) [21–25] and functional near-infrared spectroscopy (fNIRS) [26]. The results demonstrated the capability in evaluating stereoscopic recovery. However, those methods required expensive equipment and inconvenient operations, especially unsuitable for children. Besides, the abovementioned methods were incapable of monitoring real-time neurophysiological changes during training. To solve the problem, electroencephalogram (EEG) has also been used in the relevant study. For example, Deng et al. assessed binocular processing differences following perceptual learning in adult anisometropic patients using steady-state visual evoked potential [27].

In our study, we developed a dichoptic stimulation system based on the push-pull protocol. The applicability of EEG to assess the training effect was investigated. The possibility of using EEG as a biomarker to predict the acute training effect has been discussed as well.

## 2. Materials and Methods

### 2.1. Subjects

In the present study, 69 children (4 to 17 years old, mean ± standard deviation: 7.1 ± 2.7 years old) were recruited from the Department of Ophthalmology of Beijing Children's Hospital, Capital Medical University from April 2017 to August 2018. Thirty-six children were diagnosed with anisometropia and amblyopia, and the other 33 were children with normal VA. All subjects and their parents were informed about the experimental protocol, and written informed consent was obtained. Our experiments were approved by the ethics committee of Beijing Children's Hospital.

### 2.2. EEG Data Acquisition and Associated Procedures

**Figure 1:**
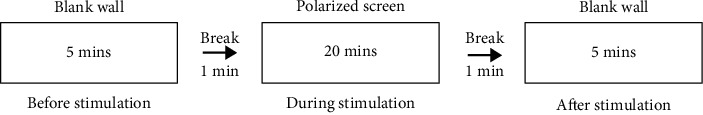
Experimental flowchart. This experiment procedure consisted of three steps: before, during, and after stimulation. In each step, the subjects were told to look at blank wall or polarized screen while recording EEG. Between each step, there is a 1-minute break.

EEG data were recorded using a NeuroScan SynAmps2 64-channel EEG amplifier (Compumedics, Victoria, Australia) with a 64-channel elastic cap (Quik-Cap, NeuroScan) at a sampling rate of 1000 Hz. The impedance of all electrodes was adjusted to less than 10 k*Ω*, and the mean values of M1 and M2 (according to the international 10-20 EEG system) were set as the reference. Before training, all subjects were well rested and stayed alert while sitting in a comfortable chair. At the beginning of training, subjects were told to look at a blank wall, and EEG signals were recorded for 5 minutes. During the stimulation, subjects placed their head in a chinrest to ensure screen alignment (the centre of the two eyes was aligned to the centre of the screen) and observed the stimuli for 20 minutes. EEG signals were recorded for 20 minutes simultaneously. We then recorded the EEG signal for 5 minutes after the visual stimulus, while the subjects looked at the blank wall again. The experimental flow chart is shown in Figure 1.

### 2.3. Dichoptic Stimulation

**Figure 2:**
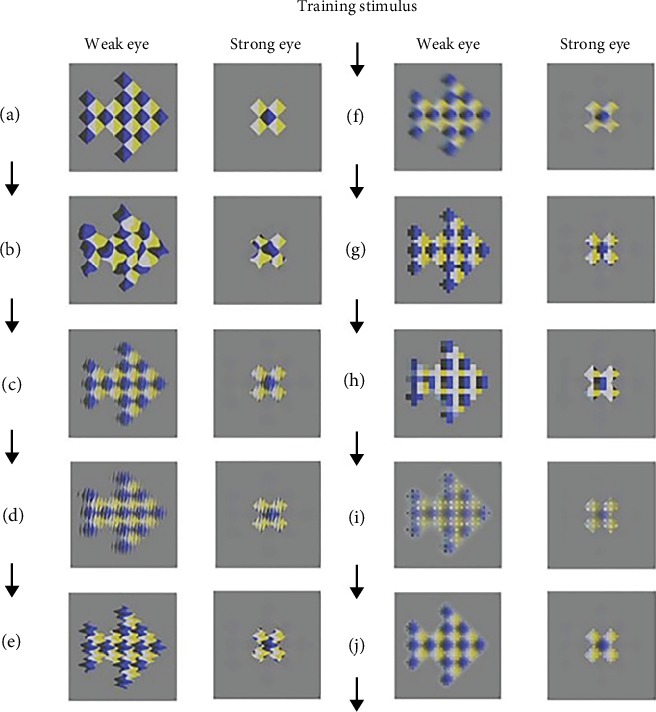
Push-pull training scenario used in the study. The initial images perceived by the two eyes are shown in (a). During the stimulation process, the images seen by both eyes were synchronously processed and switched every 500 milliseconds and looped from (a) to (j) over 5 seconds, allowing the half image viewed by the weak amblyopic eye to be perceived and the one viewed by the strong eye to be suppressed.

**Figure 3:**
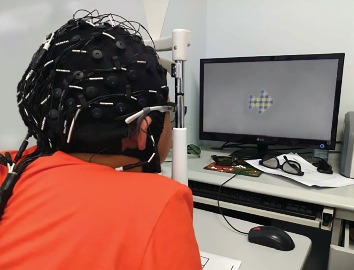
Experimental scenario. One subject was looking at a polarized screen while recording EEG.

The dichoptic stimulation was performed in a clinical assessment room with constant luminance. The experiment was conducted using a PC and an LG D2343P polarized 3D monitor (LG Electronics, Seoul, South Korea) with a resolution of 1920 × 1080 and a refreshing rate of 120 Hz. All subjects wore polarized glasses and their corrective lenses. The visual stimuli were developed using the push-pull perceptual learning protocol and presented at an observation distance of 80 cm, with a background brightness of 35 candelas *per* square meter (cd/m^2^). During the stimulation, the rivalling half image to the amblyopic eye was perceived (push), while the half image to the strong eye was perceptually suppressed (pull) (see Figure 2). The image size for the strong eye was 200 × 200 pixels, and the image size for the weak eye had four levels: 200 × 200, 400 × 400, 600 × 600, and 800 × 800 pixels. The contrast ratio for weak eye was set as 100% during the stimulation, while 5% to 50% for the strong eye. The selection of the image size and contrast ratio depended on the extent of the VA disparity. For normal VA subjects, the image size for both eyes was set at 200 × 200 pixels, and contrast ratio was set at 100%.  Figure 3 shows an experimental scenario in which a subject was looking at the stimulus image on the screen, while the EEG was being recorded.

### 2.4. EEG Data Analysis and Statistical Analysis

The raw EEG signals were processed offline using Curry 7.0 (Compumedics, Victoria, Australia) and MATLAB (MathWorks, Natick, MA). The preprocessing of the raw EEG data included the use of band-pass filters (0.1-30 Hz), rereferencing (the mastoids are chosen as reference electrodes), and independent component analysis (ICA) for artefact removal (including eye movements, temporal muscle activity, and linear noise). The procedures were realised by EEGLAB [28]. We calculated the power spectral density (PSD) of each electrode for three different states, i.e., before, during, and after stimulation, using Welch method [29]. Moreover, four frequency bands were analyzed in the study, i.e., delta (0.1 to 4 Hz), theta (4 to 8 Hz), alpha (8 to 14 Hz), and beta (14 to 30 Hz). To reduce the impact of individual differences, the absolute PSD in each frequency band was normalized by dividing by the total PSD to obtain the relative PSD, which was used for statistical analysis.

A two-way analysis of variance (ANOVA) was performed (*per* channel and *per* frequency band) to evaluate the resulting PSD changes with the factors of VA disparity and stimulation state. VA was converted to the logarithm of the minimum angle of resolution (LogMAR). Accordingly, the subjects were divided into three levels: 0 to 0.2 (2 lines in the visual chart of LogMAR) for mild VA disparity, 0.2 to 0.4 (2 to 4 lines) for moderate VA disparity, and above 0.4 for severe VA disparity (exceeding 4 lines). There were 10 subjects with mild VA disparity, 13 subjects with moderate VA disparity, and 13 with severe VA disparity. The three stimulation states were before, during, and after stimulation. In order to assess the effect relating to different ages, we also divided 69 subjects into two groups, i.e., 16 patients plus 18 normal VA subjects in the group below 7 years old and 20 patients plus 15 normal VA subjects in the group exceeding 7 years old. We performed independent *t* tests for the normal VA subjects and the patients, respectively, to assess the between-group difference.

Moreover, it would be of great interest to investigate the correlation between the EEG change during stimulation and the treatment effect, because it can provide insight on predicting the individual recovery effect which enabled the optimization of the training contents. The statistical experiments were designed for the 36 children with anisometropia and amblyopia. Correlation between recovery effect (calculated by subtracting the poststimulation VA disparity from the prestimulation VA disparity) and the change of relative PSD was investigated. The analyses were conducted per channel and per band.

The statistical analyses were performed using SPSS 25.0 (IBM; Armonk, NY).

## 3. Results

**Figure 4:**
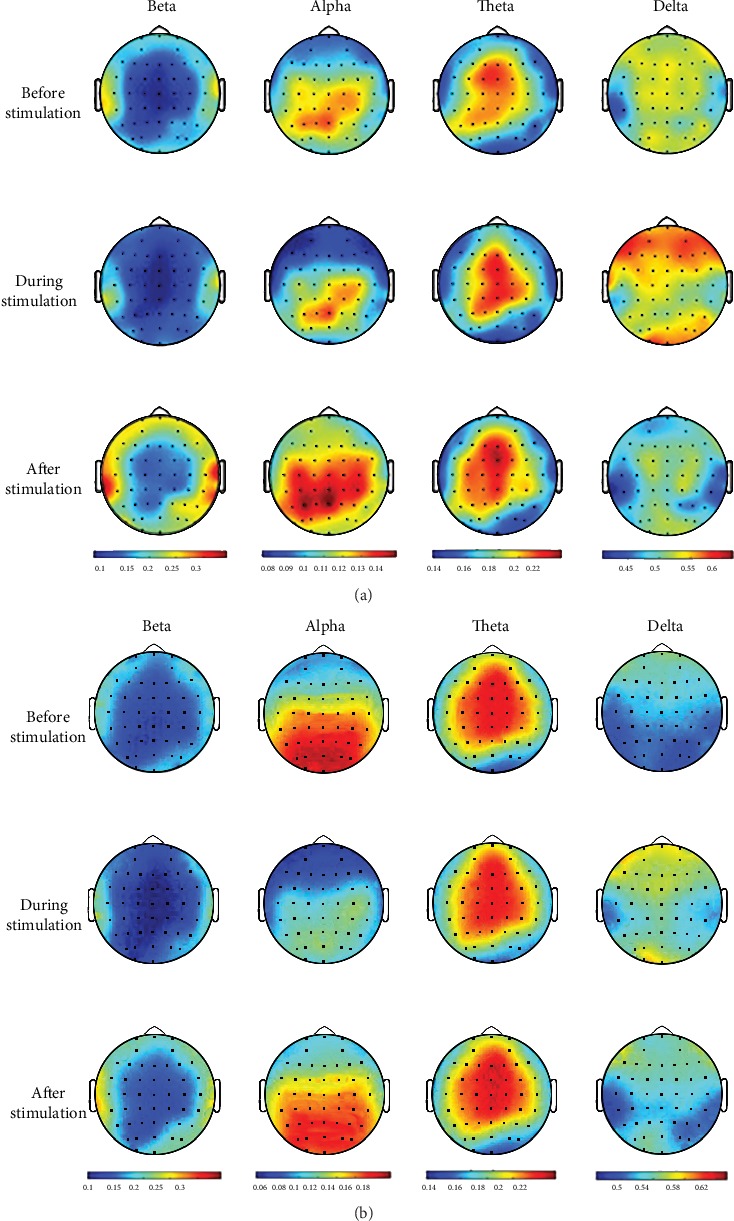
Mean EEG topographic maps over 36 anisometropic subjects (a) and 33 normal VA subjects (b). Each column represents the distribution of the averaged relative PSDs of the EEGs for the four frequency bands, while each row represents different stimulation states (before, during, and after stimulation).

Figure 4 illustrates the EEG topographic maps averaged across all 36 amblyopia subjects and 33 normal VA subjects in four frequency bands. Two-way ANOVA yielded significant differences for the interaction effect of VA disparity and stimulation state in the beta pattern, TP7, *p* = 0.004, *F* (4, 198) = 4.016. There were no other channels or frequency bands with significant differences for the interaction effect. For the main effect of VA disparity, we found significant differences in the delta band (C6, T8, C1, CZ, CP1, CPZ, *p* < 0.01) and the theta band (F7, F5, F6, F8, TP7, T8, *p* < 0.01). For the main effect of stimulation state, significant differences were found in the beta (C1, C6, P1, P6, T7, P8, TP7, TP8, O1, O2, OZ, *p* < 0.01) and delta bands (FP1, FPZ, FP2, AF3, AF4, F7, F5, F6, F8, TP7, T8, *p* < 0.01). We used the least significant difference (LSD) for the post hoc test. In terms of after vs. before stimulation, the post hoc test yielded significant differences in the beta bands across six channels: T7 (*p* = 0.009), P8 (*p* = 0.007), TP8 (*p* = 0.008), O1 (*p* = 0.004), OZ (*p* = 0.001), and O2 (*p* = 0.007). There was no significant change after vs. before stimulation in the delta frequency bands. We also found significant differences in the beta and delta rhythms after vs. during stimulation (beta: all channels; delta: FP1, FPZ, FP2, AF3, AF4, F7, F5, F6, *p* < 0.01), including occipital areas and the temporal lobes.

To compare the EEG differences between normal subjects and subjects with anisometropia, we performed *t* tests for before and after the stimulation on the relative PSDs of the four frequency bands of the 33 normal VA subjects and the 36 anisometropic subjects (per channel). The *t* test results from the normal VA subjects revealed no significant differences across all channels and all frequency bands, but the results from the anisometropic patients revealed significant differences in the beta frequency PSDs on 25 channels (FT7, FC5, FC3, T7, C5, C3, C4, C6, T8, TP7, CP5, CP3, CP4, CP6, TP8, P5, P3, P1, P4, P6, P8, O1, OZ, O2). The anisometropic subjects had no significant differences in any of the channels in the alpha, theta, or delta bands. The *t* tests for between-group difference showed no significant changes from the normal VA subjects and the patients in the two age groups.

**Figure 5:**
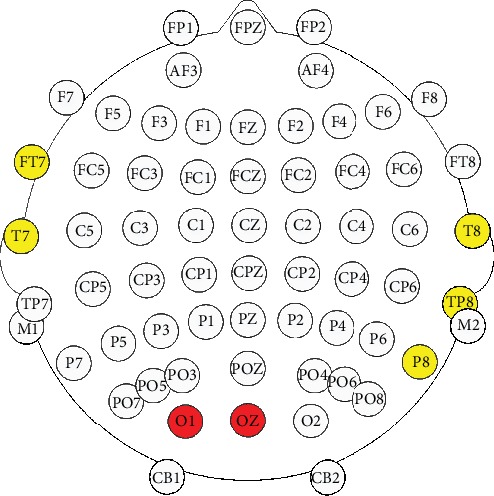
EEG channels showed significant correlation between relative PSD change and training effect. Red colour indicates the channels with correlation coefficient beyond 0.8, while yellow colour indicates the channels with correlation coefficient between 0.5 and 0.8.

The correlation analysis showed that high correlation (*r* > 0.8) for two channels at occipital region (O1 and OZ). Five channels at bilateral middle temporal regions (FT7, T8, T7, P8, and Tp8) showed correlation coefficients between 0.5 and 0.8. The detected significant correlation was found at beta band. The channels are shown in Figure 5.

## 4. Discussion

After visual training, amblyopic patients showed significantly changed beta band PSD at occipital and middle temporal regions. In contrast, no difference was detected between before and after training from normal VA subjects. The finding revealed that the stimulation based on the push-pull protocol had limited effects on normal VA subjects. The comparison showed no difference between the two age groups, which may indicate that the applied stimulus pattern was not age-dependent.

Amblyopia is believed to result from a dysfunction of the cortical developmental processes [23, 24], although the exact extent of this visual cortical deficit is currently unknown. As a result, functional neuroimaging techniques have been used to study the changes at the neurophysiological level after treatment [25]. For example, Yang et al. studied blood oxygen level-dependent activity in amblyopia patients before and after levodopa treatment [24]. Similarly, Gupta et al. studied hemodynamic changes in individuals with strabismic amblyopia using fMRI and diffusion tensor imaging before and after eye patching [25]. In addition, Iwata et al. used fNIRS to assess the differences in oxygenated haemoglobin concentration changes between binocular and monocular treatment [26]. Their results revealed that during or after stimulation, the occipital region demonstrated neurophysiological differences, such as an elevated number of active voxels or blood oxygen levels, resulting in improved stereopsis after stimulations. Numerous studies have shown that during visual stimulation, beta band or gamma band power increases in occidental-parietal brain regions [27, 28]. The increase of high-frequency power was associated with enhanced neuronal discharge activity [30], which was likely linked to the increase of blood-oxygen concentration. The change in the delta band may be associated with fatigue. In all, the EEG results were consistent with reports using fNIRS and fMRI. In view of its mobility and convenience, EEG was advantageous in evaluating the acute effect of stereoscopic training.

There was a significant difference in beta power spectral density in the primary visual cortex, and there was also a significant difference in the middle temporal lobe region, where MT was located [31]. MT was a specific region of the dorsal extrastriata processing stream, which was highly motion sensitive and can integrate local motion signals into a coherent motion representation [32, 33]. In fact, converging evidence from human psychophysics and animal neurophysiology has indicated that stereopsis was associated with abnormal MT function. Our study showed that after visual stimulation, the temporal lobe area of the anisometropic subjects was active (compared to before stimulation), while that of the normal subjects was not, suggesting that MT played an important role in the formation of anisometropia. Dichoptic stimulation in our study was able to stimulate MT as well as the visual cortex, thus possibly creating a higher chance of restoring the stereo sensitivity of anisometropia.

Analysis focusing on patients with anisometropia and amblyopia clearly demonstrated that the relative PSD change during training and the training effect correlated significantly along the dorsal extrastriata processing stream. The most intense correlation was found at area V1, which was essential to the conscious processing of visual stimuli [34]. In addition, damage to V1 may lead to disruption or even loss specific aspects of vision (e.g., depth perception [35]). The strong correlation between the PSD change and the posttraining effect along the dorsal visual stream revealed that these regions may closely associate with the acute treatment effect. It also indicated the possibility to use the EEG data from this area as an indicator for choosing the desired training contents. As such, it has the potential to act as a biomarker for predicting the stimulating effect and for adjusting the individual training plan (the stimulation length or the content). The features derived from the EEG on these channels may also be employed as a classifier to identify the appropriate stimulation method for the patients, which would be investigated our further studies when more subjects were recruited.

The limitation of the study was the limit number of the subjects under evaluation. In addition, we did not track their long-term training effect due to the mobility of the subjects.

## 5. Conclusions

In our study, we focused on the stereoscopic recovery of amblyopic patients. We designed a dichoptic stimulation system adopted from push-pull perceptual learning using polarized glasses, performed training on 33 normal VA children and 36 anisometropic children, and evaluated its acute effects through EEG. After a 20-min dichoptic stimulation, we detected enhanced beta rhythm PSD after stimulation in the visual cortex and MT of the anisometropic subjects. Our study provided evidence indicating that dichoptic training was able to stimulate MT and the primary visual cortex, and EEG acquired in those regions has potential applications in evaluating the acute effect of stereoscopic training. The study also discussed the possibility of using the EEG signal as biomarker to predict the acute training effect. In future studies, we will enroll more subjects and investigate the use of this EEG feature to optimizing the individual training plan.

## Data Availability

The EEG data used to support the findings of this study are available from the corresponding author upon request.
